# Dextran sulphate-induced tau assemblies cause endogenous tau aggregation and propagation in wild-type mice

**DOI:** 10.1093/braincomms/fcaa091

**Published:** 2020-07-08

**Authors:** Masami Masuda-Suzukake, Genjiro Suzuki, Masato Hosokawa, Takashi Nonaka, Michel Goedert, Masato Hasegawa

**Affiliations:** f1 Dementia Research Project, Tokyo Metropolitan Institute of Medical Science, Setagaya-ku, Tokyo 156-8506, Japan; f2 MRC Laboratory of Molecular Biology, Cambridge CB2 0QH, UK

**Keywords:** Tau, propagation, dextran sulphate, wild-type mice

## Abstract

Accumulation of assembled tau protein in the central nervous system is characteristic of Alzheimer’s disease and several other neurodegenerative diseases, called tauopathies. Recent studies have revealed that propagation of assembled tau is key to understanding the pathological mechanisms of these diseases. Mouse models of tau propagation are established by injecting human-derived tau seeds intracerebrally; nevertheless, these have a limitation in terms of regulation of availability. To date, no study has shown that synthetic assembled tau induce tau propagation in non-transgenic mice. Here we confirm that dextran sulphate, a sulphated glycosaminoglycan, induces the assembly of recombinant tau protein into filaments *in vitro*. As compared to tau filaments induced by heparin, those induced by dextran sulphate showed higher thioflavin T fluorescence and lower resistance to guanidine hydrochloride, which suggests that the two types of filaments have distinct conformational features. Unlike other synthetic filament seeds, intracerebral injection of dextran sulphate-induced assemblies of recombinant tau caused aggregation of endogenous murine tau in wild-type mice. AT8-positive tau was present at the injection site 1 month after injection, from where it spread to anatomically connected regions. Induced tau assemblies were also stained by anti-tau antibodies AT100, AT180, 12E8, PHF1, anti-pS396 and anti-pS422. They were thioflavin- and Gallyas-Braak silver-positive, indicative of amyloid. In biochemical analyses, accumulated sarkosyl-insoluble and hyperphosphorylated tau was observed in the injected mice. In conclusion, we revealed that intracerebral injection of synthetic full-length wild-type tau seeds prepared in the presence of dextran sulphate caused tau propagation in non-transgenic mice. These findings establish that propagation of tau assemblies does not require tau to be either mutant and/or overexpressed.

## Teaser

We demonstrate that tau filaments assembled from recombinant tau protein and dextran sulphate differ from those assembled using heparin. Intracerebral injection of tau filaments induced by dextran sulphate into wild-type mice caused seeding of endogenous murine tau, followed by spreading to distinct brain areas in a time-dependent manner.

## Graphical Abstract

**Graphical Abstract:**
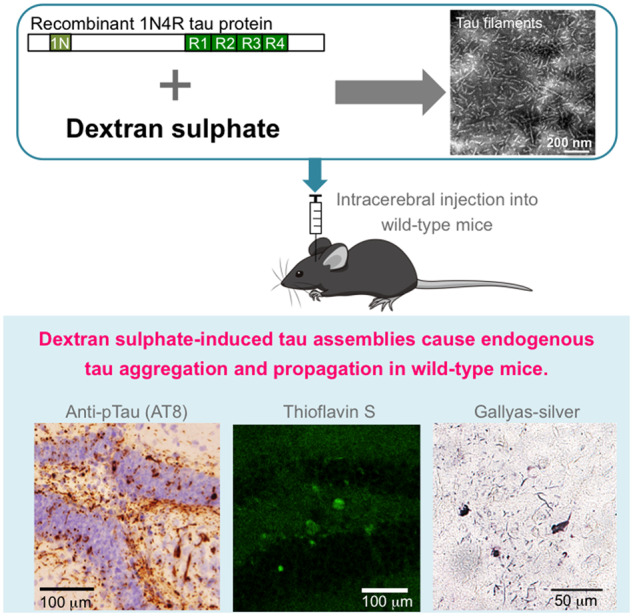

## Introduction

Tauopathies are progressive neurodegenerative diseases with accumulation of assembled tau protein in the neuronal and glial cells of the nervous system. They include Alzheimer’s disease, argyrophilic grain disease, corticobasal degeneration, chronic traumatic encephalopathy, globular glial tauopathy, Pick’s disease and progressive supranuclear palsy (Goedert *et al.*, 2017). Most cases of tauopathy are sporadic and there are no approved disease-modifying therapies.

Tau is a microtubule-associated protein that is expressed predominantly in the axons of the nerve cell. In the adult human brain, six isoforms of tau are found based on the presence of 0, 1 or 2 N-terminal inserts and three (3R) or four (4R) microtubule-binding repeats (Goedert *et al.*, 1989). Some diseases show isoform-specific accumulation of tau: 3R isoforms in Pick’s disease, 4R isoforms in argyrophilic grain disease, corticobasal degeneration, globular glial tauopathy, progressive supranuclear palsy and 3 R + 4R isoforms in Alzheimer’s disease and chronic traumatic encephalopathy. Soluble tau is natively unfolded, whereas assembled tau forms amyloid filaments with β-sheet structure (Berriman *et al.*, 2003; Fitzpatrick *et al.*, 2017). Assembled tau is hyperphosphorylated and partially ubiquitinated in tauopathy brains (Hasegawa *et al.*, 1992; Morishima-Kawashima *et al.*, 1993). Understanding how abundant tau inclusions form in the brain is of paramount importance.

Tau pathology develops stereotypically and correlates with the clinical staging of sporadic Alzheimer’s disease (Braak and Braak, 1991), which suggests that the spreading of insoluble tau may be involved in neurodegeneration and disease progression. Intracerebral injection of inclusion-bearing brain homogenates obtained from transgenic mice led to formation of tau inclusions in mice transgenic for wild-type human tau, and the spreading of pathology to distant brain areas (Clavaguera *et al.*, 2009). Similar findings were reported when brain extracts from human tauopathies were injected (Clavaguera *et al.*, 2013; Ahmed *et al.*, 2014; Boluda *et al.*, 2015). In wild-type mice, intracerebral injection of human tauopathy brain extracts also induced pathology (Lasagna-Reeves *et al.*, 2012; Audouard *et al.*, 2016; Guo *et al.*, 2016; Narasimhan *et al.*, 2017; Ferrer *et al.*, 2019*a*, *b*; Vergara *et al.*, 2019). In contrast, tau filaments assembled using recombinant 4R tau and heparin (HP) failed to induce pathology after intracerebral injection into wild-type mice (Guo *et al.*, 2016).

Here we show that tau filaments assembled from recombinant murine and human 4R tau and dextran sulphate (DS) differ from those assembled using HP. Upon intracerebral injection into wild-type mice, 1N4R tau filaments induced by DS seeded aggregation of endogenous murine tau, followed by spreading to distinct brain areas.

## Materials and methods

### Expression and purification of recombinant tau

Full-length human and murine 1N4R tau were prepared as previously described (Hasegawa *et al.*, 1998; Taniguchi *et al.*, 2005). Briefly, tau proteins were expressed in *Escherichia coli* BL21 (DE3) cells, and after centrifugation of the cell suspension, pellets were lysed in buffer A [50 mM PIPES, pH 6.9, 1 mM EGTA, 1 mM dithiothreitol (DTT) and 0.5 mM phenylmethylsulfonyl fluoride] and sonicated on ice. Lysates were centrifuged at 21 000 g for 15 min at 4°C, and supernatants were boiled in the presence of 1% 2-mercaptopethanol for 5 min followed by centrifugation at 21 000 g for 15 min. Heat-stable fractions were loaded onto an SP-Sepharose ion-exchange chromatography column (GE Healthcare) and tau protein was eluted with 0.35 M NaCl in buffer A. After precipitation by ammonium sulphate (50% saturation), tau protein was dialysed against 30 mM Tris-HCl, pH 7.5. After ultracentrifugation at 113 000 g for 20 min, the supernatant was used as soluble monomeric tau. Protein concentration was determined based on absorbance at 215 nm by reverse-phase high-pressure liquid chromatography with Aquapore RP300 column (PerkinElmer) (Taniguchi *et al.*, 2005) and double-checked using a NanoDrop 2000 spectrophotometer (molecular weight: 42 966 for human 1N4R and 41 840 for mouse 1N4R; molar extinction coefficient: 7450 for human 1N4R and 5960 for mouse 1N4R) (Thermo).

### Tau filament assembly

Purified tau (15 µM) was incubated with 40 µg/ml HP sodium (#411210010, ACROS organics) or DS sodium salt from *Leuconostoc* spp. M_r_ 5,000 (#31404, SIGMA) at 37°C in buffer B (30 mM Tris-HCl, pH 7.5, 5 mM DTT, 0.1% sodium azide) with shaking at 200 rpm for 7 days. For evaluation of thioflavin fluorescence, 10 µl sample was added to 90 µl thioflavin T (10 µM; Tokyo Chemical Industry) and the mixture was incubated at room temperature for 15 min; fluorescence intensity (excitation: 450 nm; emission: 480 nm) was measured by a Varioskan microplate reader (Thermo). To monitor sarkosyl-insolubility, 10-µl sample was added to 40 µl of 1% sarkosyl in 30 mM Tris-HCl, pH 7.5 and incubated at room temperature for 15 min. The samples were centrifuged at 100 000 g for 20 min at 25°C. The supernatants were kept as sarkosyl-soluble fractions. Following resuspension in sample buffer, the pellets were analysed by sodium dodecyl sulphate-polyacrylamide gel electrophoresis and Coomassie Brilliant Blue staining. Band densities were quantified using Image J software. Three independent experiments were performed using three separate batches of the recombinant tau protein.

### Tau seeds

Human or mouse tau (90 µM) was incubated in buffer B with 200 µg/ml HP or DS at 37°C for 7 days. The mixture was centrifuged at 113 000 g for 20 min, and the pellets were washed with saline and centrifuged again. The pellets (tau seeds) were resuspended in saline and stored at −80°C. To determine the concentrations of insoluble tau, proteins were disaggregated with 6 M guanidine hydrochloride and submitted to reverse-phase high-pressure liquid chromatography.

### Transmission electron microscopy

Tau seeds were spotted onto a carbon-coated grid (NISSHIN EM) and negatively stained with 2% phosphotungstate. Observation was performed using a JEM-1400Plus electron microscope (JEOL).

### Guanidine hydrochloride disaggregation

Guanidine disaggregation assay was performed as previously described (Falcon *et al.*, 2015). Briefly, 20 µM tau seeds were incubated with 0.5–6 M guanidine hydrochloride (GuHCl) in 10 mM phosphate buffer, pH 6.8, for 1 h at room temperature. The samples were centrifuged at 100 000 g at 25°C for 20 min. The insoluble pellets were resuspended in sample buffer and analysed by sodium dodecyl sulphate-polyacrylamide gel electrophoresis and Coomassie Brilliant Blue staining.

### Stereotactic injection

Tau seeds were sonicated for 30 s twice at 30% intensity using a Sonifier SFX250 cup horn sonicator (BRANSON) before use. Male and female C57BL/6J wild-type (Japan SLC) or *MAPT* knockout mice (Dawson *et al.*, 2001) (Jackson Laboratory) of age 4–6 months were used. Animals were group housed (four or five animals per cage) with free access to food and water. Anaesthesia was performed using isoflurane and 5 µl tau seeds (90 µM) were injected unilaterally into the hippocampus (AP: −2.5 mm; ML: 2 mm; DV: −2.3 mm from the bregma and dura) or striatum (AP: 0.2 mm; ML: 2 mm; DV: −2.6 mm). For tissue collection, mice were deeply anaesthetized by intraperitoneal injection of pentobarbital and perfused with phosphate-buffered saline. For immunohistochemistry (the number of mice used is shown in Table 1), the brains were fixed in 10% formalin neutral buffer solution (Wako); for biochemistry, the brains were snap frozen and stored at −80°C (*n* = 2/each time point). All experiments were carried out in agreement with the Guidelines for Proper Conduct of Animal Experiments (Science Council of Japan) and the ARRIVE guidelines, and all experimental protocols were approved by the Animal Care and Use Committee of the Tokyo Metropolitan Institute of Medical Science.

**Table 1:** Summary of intracerebral injection experiments

Strain	Injection materials	Injection sites	Time from injection			
			1 month	3 months	6 months	12 months
WT mice (C57BL/6J)	DS-induced mouse tau seeds	Hippo	4/4	4/4	4/4	
	DS-induced mouse tau seeds	Str	4/4	4/4	4/4	
	DS-induced human tau seeds	Hippo	4/4	4/4	4/4	
	Soluble mouse tau	Hippo			0/5	
*MAPT* KO mice (B6.129-Mapt <tm1Hnd>/J)	DS-induced mouse tau seeds	Str		0/2		0/2

Number of mice with AT8-positive pathology/number of mice used is shown.

Hippo = hippocampus; Str = striatum.

### Immunohistochemistry

Fixed brains were sectioned at 50-µm thickness using a VT1200 vibratome (Leica). Free-floating sections were mounted on glass slides and processed for antigen retrieval by heating at 100°C in 0.1 M sodium citrate buffer, pH 6.0, for 10 min and by immersing in 95% formic acid for 10 min (Masuda-Suzukake *et al.*, 2014). Sections were then treated with 3% hydrogen peroxide in methanol to inactivate endogenous peroxidases, permeabilized with 0.5% Triton X-100 in phosphate-buffered saline and blocked with 0.3% Triton X-100 in 10% foetal calf serum in phosphate-buffered saline. Primary antibodies (Supplementary Table 1) in blocking buffer were incubated at room temperature overnight; subsequently, biotin-conjugated secondary antibodies (1:500; Vector) were added. Sections were then incubated with Vectastain ABC kit (Vector) and developed using 0.4 mg/ml 3,3ʹ-diaminobenzidine (Sigma). Following counterstaining with haematoxylin (Muto Chemicals), the sections were coverslipped.

Quantitative analyses were performed on the three adjacent sections of the injection sites and the major brain regions of propagation. For the mice injected into the hippocampus, hippocampus (2.5 mm posterior to bregma) and mammillary nucleus (2.8 mm posterior to bregma) were analysed. For the mice injected into the striatum, striatum and corpus callosum (0.2 mm anterior to bregma), and substantia nigra (3.1 mm posterior to bregma) were analysed. Images were taken by BZ-X710 microscope (Keyence) and AT8-positive area in the selected region was measured by BZ-X analyser software (Keyence).

### Immunofluorescence

For immunofluorescence, Alexa488- or Alexa568-conjugated secondary antibodies (1:1000; Thermo) were used. After staining with Hoechst 33342 (Life Technologies), the sections were coverslipped using Vectashield (Vector). Images were acquired using BZ-X710 fluorescence microscope.

### Gallyas-Braak silver

Brain sections were mounted on APS^™^-coated glass slides (Matsunami glass) and air-dried. Delipidation was achieved with increasing concentrations of ethanol and xylene, followed by washes in distilled water. Silver staining was conducted according to the Gallyas-Braak method (Gallyas, 1971; Braak *et al.*, 1988; Uchihara, 2007).

### Thioflavin S staining

Brain sections were stained with 0.001% thioflavin S (Polysciences) in 20% ethanol for 30 min, followed by washes in 20% ethanol and water. Images were acquired using LSM780 confocal microscope (Zeiss).

### Biochemical analysis

Sarkosyl extraction of the mouse brains was performed as previously described (Masuda-Suzukake *et al.*, 2013). The brains were divided into two hemispheres along the longitudinal fissure and homogenised in buffer C (10% sucrose, 10 mM Tris-HCl, pH 7.5, 0.8 M NaCl and 1 mM EGTA) using a Polytron. The homogenates were centrifuged at 100 000 g for 30 min, and the supernatants obtained were kept as the soluble fractions. The pellets were homogenized in 20 volumes buffer C containing 1% Triton X-100, followed by incubation at 37°C for 30 min. After centrifugation, the samples were homogenized in buffer C containing 1% sarkosyl and incubated at 37°C for 30 min. The samples were then centrifuged at 100 000 g for 30 min, and the pellets were resuspended in 30 mM Tris-HCl, pH 7.5, which yielded the sarkosyl-insoluble fractions. Samples were run on 10% sodium dodecyl sulphate-polyacrylamide gel electrophoresis and transferred on polyvinylidene fluoride membranes. The membranes were blocked with 5% skim milk in 0.05% Tween20 in phosphate-buffered saline and incubated with primary antibodies (Supplementary Table 1). Following incubation with horseradish peroxidase-conjugated secondary antibodies (1:10 000; Bio-Rad) for 1 h, signal was developed using Supersignal West Dura extended duration Substrate (Thermo) and detected using ImageQuant LAS 4000mini (General Electric).

### Statistical analyses

Data are expressed as mean ± standard error of the mean (SEM). Statistical comparisons were performed with unpaired *t*-test or one-way ANOVA with Tukey’s *post hoc* test using GraphPad Prism 8 software.

### Data availability

The datasets used and/or analysed during the current study are available from the corresponding author on reasonable request.

## Results

### Recombinant tau assembled using DS has different properties from that assembled with HP

**Figure 1:**
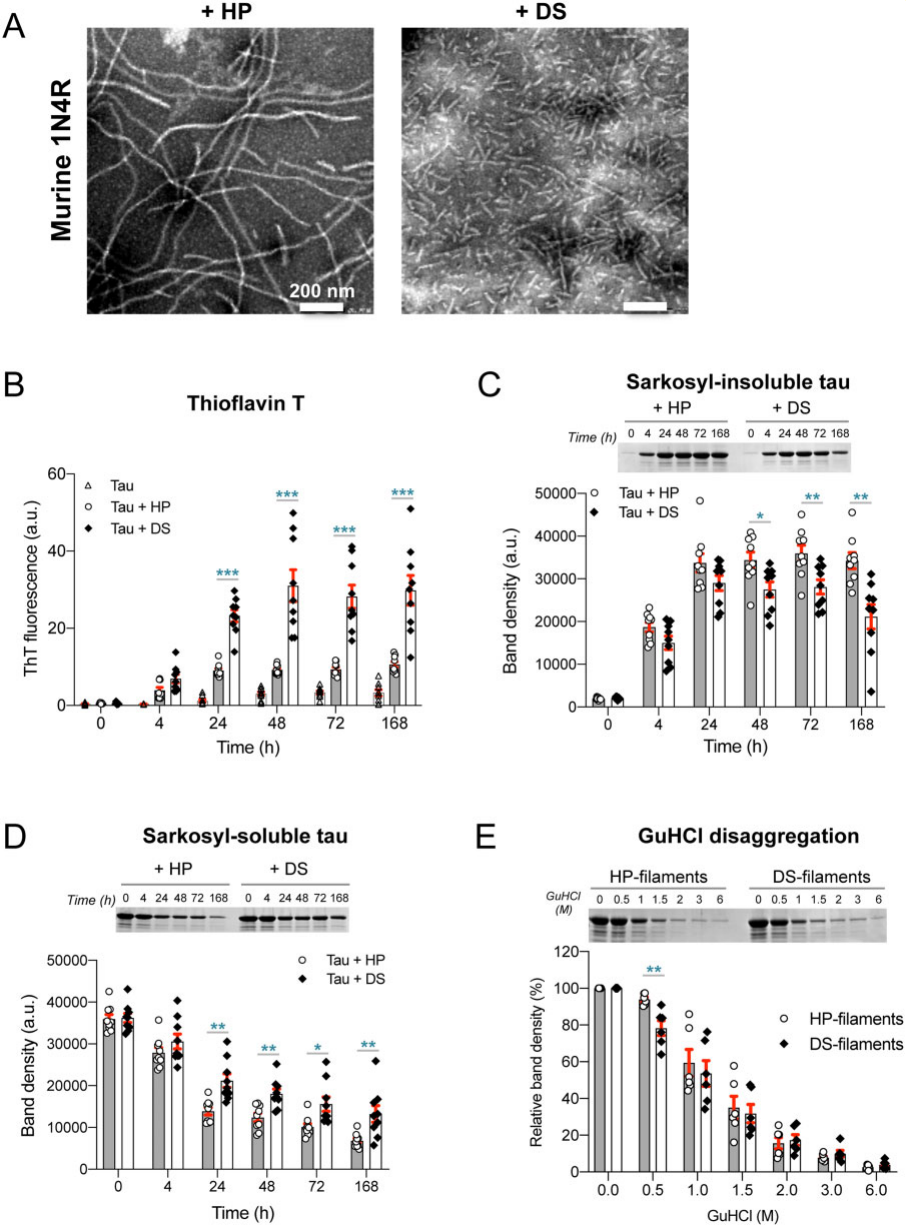
**Different properties of HP- and DS-induced murine tau filaments.** (**A**) Negative-stain electron micrographs of recombinant murine 1N4R tau following incubation with HP or DS. Scale bar, 200 nm. (**B**) Kinetics of tau assembly in the presence of HP or DS based on thioflavin T fluorescence (*n* = 9). (**C**, **D**) Formation of sarkosyl-insoluble and -soluble tau in the presence of HP or DS. The proteins were separated on sodium dodecyl sulphate-polyacrylamide gel electrophoresis, stained with Coomassie Brilliant Blue and quantified by ImageJ software (*n* = 9). Full-length gel images are shown in Supplementary Fig. 6A and B. (*E*) GuHCl disaggregation of preformed murine tau filaments. HP-induced tau filaments are more resistant to 0.5 M GuHCl than DS-induced tau filaments (*n* = 6). Full-length gel image is shown in Supplementary Fig. 6C. Mean and S.E.M. are shown in the graph. Statistical analysis was performed with unpaired t-test (*P < 0.05; **P < 0.01; ***P < 0.001).

Murine 1N4R tau was incubated with DS or HP and both cofactors induced the formation of large number of filaments (Fig. 1A). Similar to what was observed previously by negative-stain electron microscopy with 1N3R human tau (Hasegawa *et al.*, 1997), filaments formed in the presence of DS were shorter than those induced by HP. When assembly was monitored using thioflavin T, DS induced faster assembly than HP and plateaued at 3-fold higher levels of fluorescence (Fig. 1B). This was not influenced by filament lengths, since no change in thioflavin T fluorescence was obtained after fragmentation by sonication (Supplementary Fig. 1). The amount of sarkosyl-insoluble tau after 24 h was similar between DS and HP, while it was higher in HP at the later time points (Fig. 1C). Conversely, the amount of soluble tau was higher in the presence of DS (Fig. 1D). In the assessment of stability of preformed tau filament seeds under GuHCl exposure, the stability was lower for DS-induced filaments than for HP-induced seeds (Fig. 1E). Collectively, different characteristics of synthetic murine tau filaments were achieved by DS versus HP.

### DS-induced seeds cause local tau assembly and propagation in wild-type mice after injection into the hippocampus

**Figure 2:**
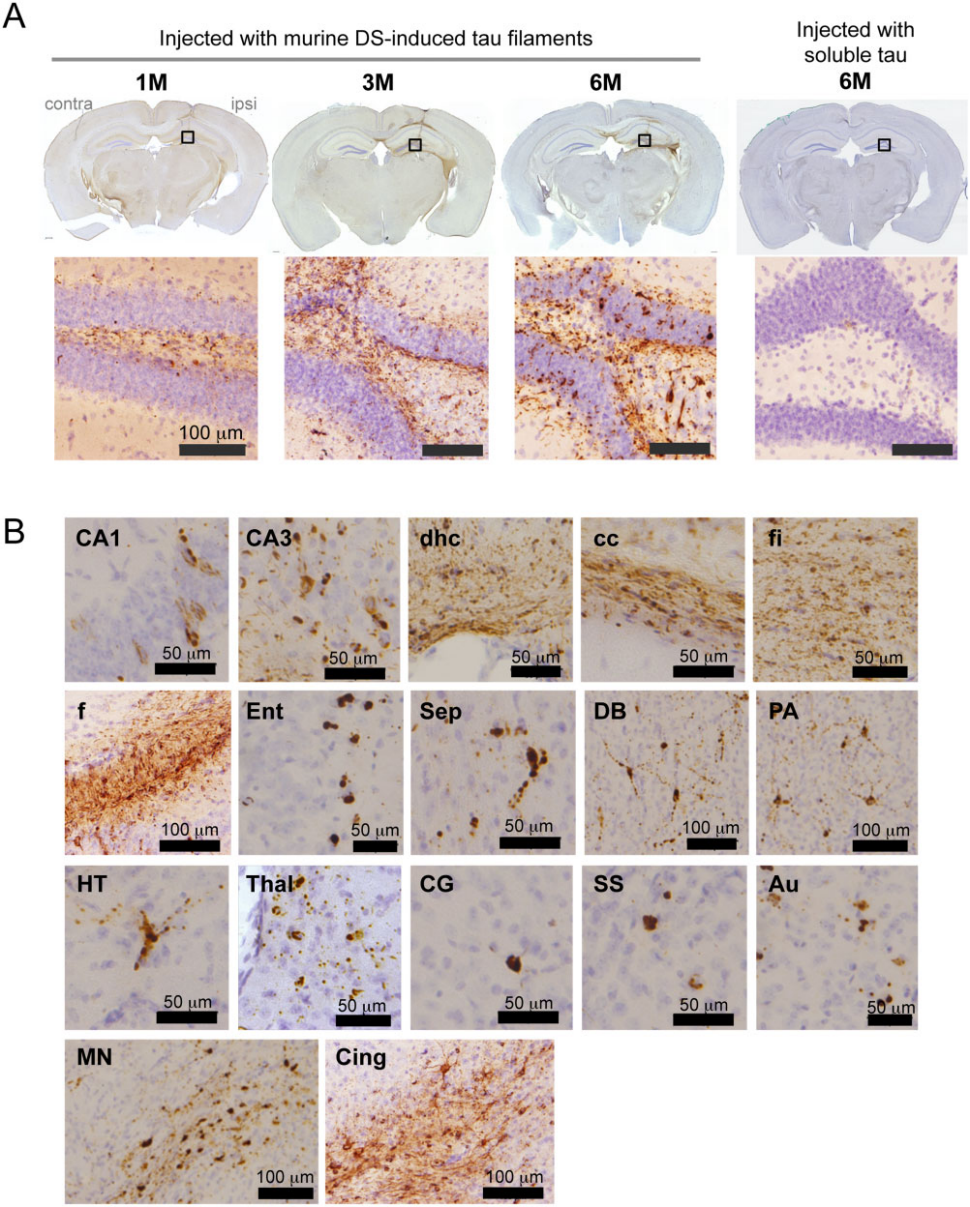
**AT8-positive staining after unilateral injection of DS-induced murine tau filaments into the hippocampus of wild-type mice.** (**A**) AT8 staining after 1, 3 and 6 months (M) of injection. Higher magnification of the boxed area is shown. No AT8-positive staining is observed at 6 M after injection of murine tau without pre-incubation with DS. Ipsi = ipsilateral (injected) hemisphere; contra = contralateral hemisphere. (**B**) AT8 staining at 6 M after injection. Au = auditory cortex; cc = corpus callosum; Cing = cingulum; CG = cingulate gyrus; DB = nucleus of the diagonal band; dhc = dorsal hippocampal commissure; Ent = entorhinal cortex; f = fornix; fi = fimbria; HT = hypothalamus; MN = mammillary nucleus; PA = pre-optic area; Sep = septal nucleus; SS = somatosensory cortex; Thal = thalamus.

**Figure 3:**
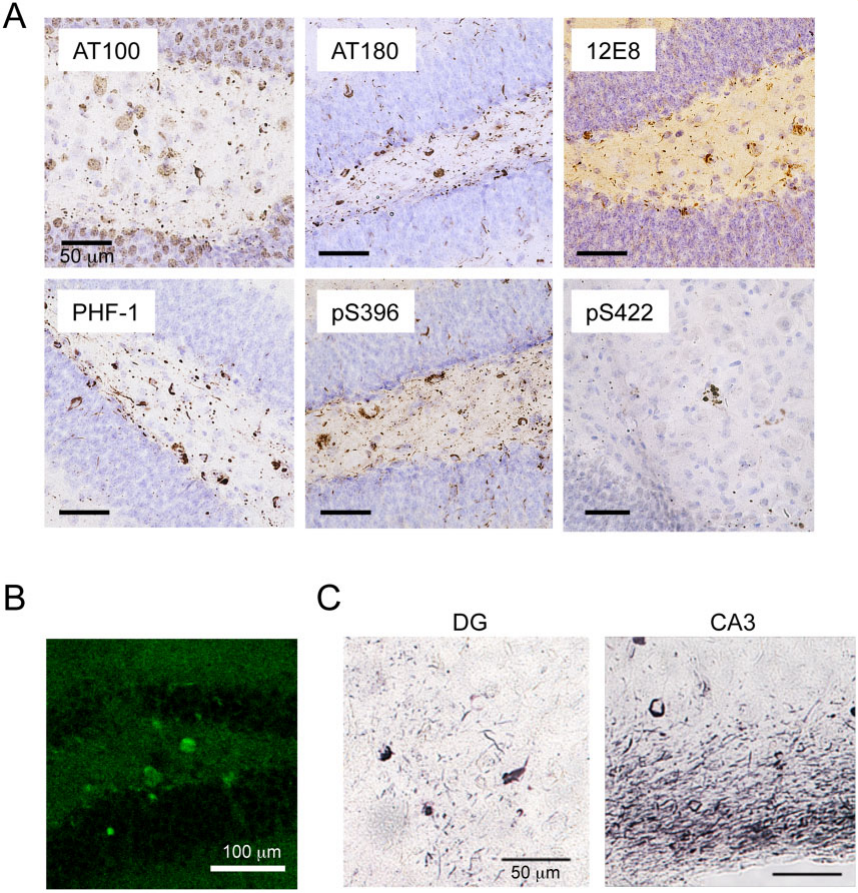
**Hyperphosphorylation and cross-β conformation (thioflavin S and Gallyas-Braak) of tau after unilateral injection of DS-induced murine tau filaments into the hippocampus of wild-type mice.** (**A**) Staining of the dentate gyrus with anti-tau antibodies AT100 (pT212/pS214/pT217), AT180 (pT231), 12E8 (pS262 and/or pS356), PHF-1 (pS396 and pS404), anti-pS396 and anti-pS422 at 6 months after injection. (**B**) Thioflavin S-positive neurons in the dentate gyrus at 6 months after injection. (**C**) Gallyas-Braak silver-positive structures in the dentate gyrus (DG) and CA3 layer of the hippocampus at 6 months after injection.

**Figure 4:**
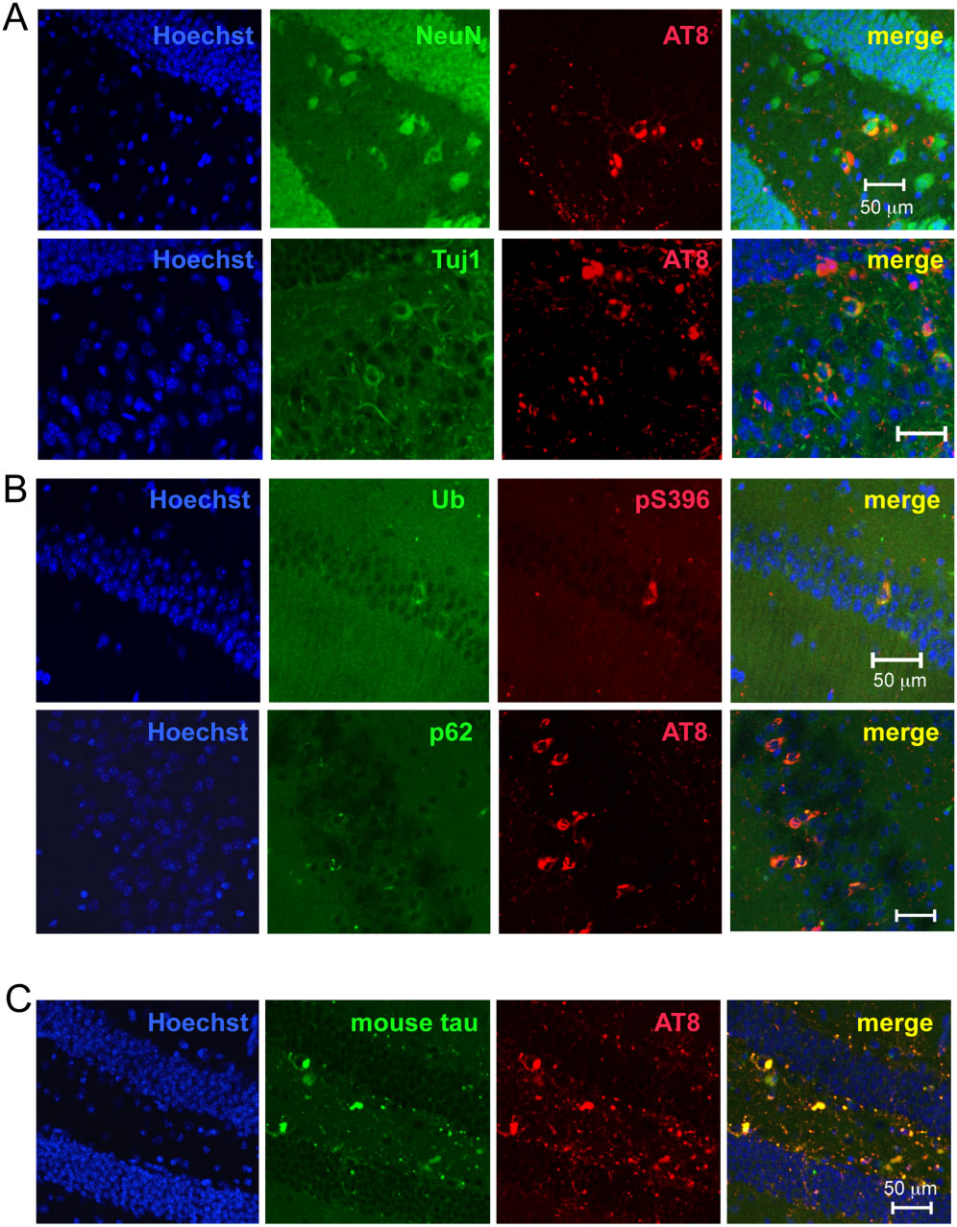
**Co-localization of phosphorylated tau pathology with neuronal markers, ubiquitin, p62 and endogenous mouse tau after unilateral injection of DS-induced tau filaments into the hippocampus of wild-type mice.** (**A**) Double staining of the dentate gyrus with AT8 and neuronal proteins antibodies NeuN and Tuj1. (**B**) Double staining with anti-pS396 or AT8 and antibodies specific for ubiquitin (in the CA1) or p62 (in the CA3). (**C**) Double staining of dentate gyrus with AT8 and an antibody specific for mouse tau in the brains injected with DS-induced human tau filaments.

In preliminary experiments, intracerebral injection of HP-induced tau seeds induced little or no tau pathology in wild-type mice (data not shown). Hence, DS-induced mouse tau assemblies were injected unilaterally into hippocampus of wild-type mice (Fig. 2). Brains were analysed 1, 3 and 6 months later. One month after injection, AT8-positive staining (pS202/pT205) was localized mainly in the polymorphic cell layer of the dentate gyrus, in the form of dot-like or short neurite-like structures (Fig. 2A). Three months after injection, AT8 staining was additionally observed in the granule cell layer of the dentate gyrus. Six months after injection, AT8 staining was denser in the nerve cell processes and some cell body staining was also present (Fig. 2A). Simultaneously, AT8 staining was observed throughout the hippocampus (Fig. 2B), and at the dorsal hippocampal commissure, corpus callosum, fimbria-fornix, entorhinal cortex, septal nucleus, nucleus of the diagonal band, pre-optic area, hypothalamus, thalamus, cingulate gyrus, somatosensory cortex, auditory cortex, mammillary nucleus and cingulum. In contrast, injection of 1N4R mouse tau without pre-incubation with DS failed to induce AT8 staining even at 6 months after injection (Fig. 2A, right). All mice injected with murine DS-induced tau seeds into hippocampus had AT8-positive pathology at all time points (Table 1). Besides AT8, tau assemblies were stained by other phosphorylation-dependent anti-tau antibodies, including AT100 (pT212/pS214/pT217), AT180 (pT231), 12E8 (pS262 and/or pS356), PHF1 (pS396/pS404), anti-pS396 and anti-pS422 (Fig. 3A). They were also positive for thioflavin S (Fig. 3B) and Gallyas-Braak silver (Fig. 3C). Double staining revealed that murine tau pathologies were mainly present in the nerve cells (Fig. 4A), where they partially co-localized with ubiquitin and p62 (Fig. 4B).

### Human tau seeds induced by DS cause tau assembly and propagation in wild-type mice after injection into the hippocampus

Next, we sought to determine if DS-induced synthetic human tau seeds also cause AT8-positive pathology and its propagation in wild-type mice.

*In vitro* analyses revealed that DS induced shorter tau filaments than HP (Supplementary Fig. 2A). Approximately 3-fold higher thioflavin T fluorescence of human 1N4R tau was observed in the presence of DS during the course of filament formation, as compared to that in the presence of HP (Supplementary Fig. 2B). The levels of sarkosyl-insoluble tau were similar between HP and DS at 4 h; while DS formed more insoluble tau at 24, 48 and 72 h; particularly at 168 h, greater level of sarkosyl-insoluble tau was observed in the presence of HP, despite 3-fold higher fluorescence in the filaments induced by DS (Supplementary Fig. 2B and C). There was no significant difference in the level of sarkosyl-soluble tau (Supplementary Fig. 2D). In the results of guanidine disaggregation assay, stability of DS-induced human tau seeds was lower at each 0.5 and 1 M concentration of GuHCl as compared to that of HP-induced seeds (Supplementary Fig. 2E). Collectively, different conformations of human tau seeds were formed by DS versus HP, consistent with the results observed in murine tau (Fig. 1).

AT8 staining was observed after injection of DS-induced 1N4R human tau seeds into the hippocampus of wild-type mice, at 1 month after injection, tau pathology was seen in the corpus callosum, and from 3 months onwards, it was present in the dentate gyrus (Supplementary Fig. 3A). At 6 months after injection, AT8-positive pathology was widely distributed (Supplementary Fig. 3B) and all mice injected with human DS-seeds had AT8-positive pathology at all time points analysed (Table 1). Double staining of AT8 with an antibody against mouse tau showed that endogenous murine tau was accumulated (Fig. 4C), indicating that DS-induced human tau filaments seeded tau in wild-type mice.

### DS-induced seeds cause local tau assembly and propagation in wild-type mice after injection into the striatum

**Figure 5:**
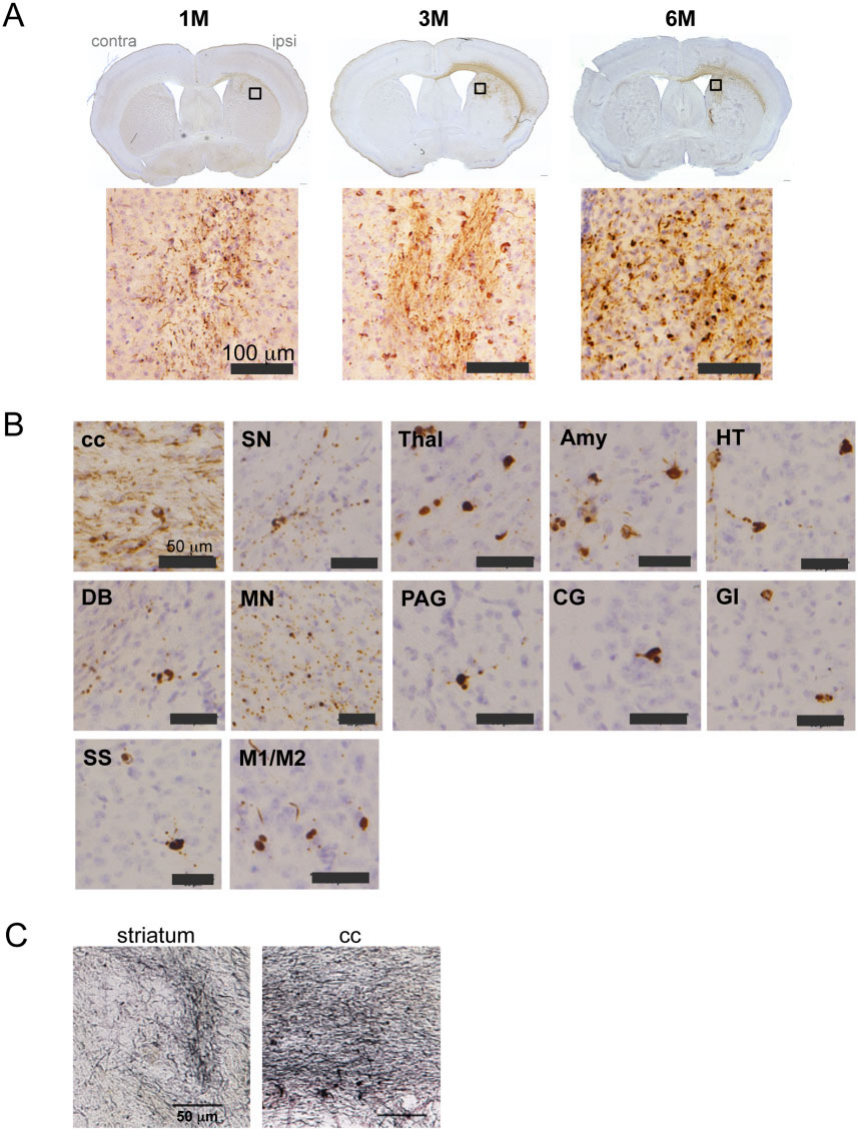
**AT8-positive staining after unilateral injection of DS-induced murine tau filaments into the striatum of wild-type mice.** (**A**) AT8 staining after 1, 3 and 6 months (M) of injection. Higher magnification of the boxed area is shown. (**B**) AT8 staining at 6 months after injection. Amy = amygdala; cc = corpus callosum; CG = cingulate gyrus; DB = nucleus of the diagonal band; GI = granular insular cortex; HT = hypothalamus; M1/M2 = primary and secondary motor cortices; MN = mammillary nucleus; PAG = periaqueductal grey; SN = substantia nigra; SS = somatosensory cortex; Thal = thalamus. (**C**) Gallyas-Braak silver-positive structures in the striatum and corpus callosum (cc) at 6 months after injection.

To better understand how tau pathologies spread, DS-induced murine tau seeds were injected into the striatum that has abundant neural connections with the other brain area. After unilateral injection of DS-induced seeds into the striatum of wild-type mice (Fig. 5A), at 1 month, AT8-positive labelling was mainly localized in the striatum and corpus callosum, where it was found in the neurites. At 3 months, it was localized in the cell bodies, and at 6 months, AT8 staining was denser (Fig. 5A). In addition to striatum and corpus callosum, AT8-positive labelling was widely distributed, including in the substantia nigra, thalamus, amygdala, hypothamalus and some cortical areas at 6 months (Fig. 5B). These structures were also positive for Gallyas-Braak silver staining (Fig. 5C). And all mice injected with murine DS-seeds into striatum showed AT8-positive pathology at 1, 3 and 6 months after injection (Table 1). In contrast, after injection of DS-induced tau assemblies into the striatum of tau knockout mice, no labelling of AT8 was observed (Supplementary Fig. 4 and Table 1).

### Distribution of tau pathology after intracerebral injection of DS-induced seeds is dependent on the injection sites

**Figure 6:**
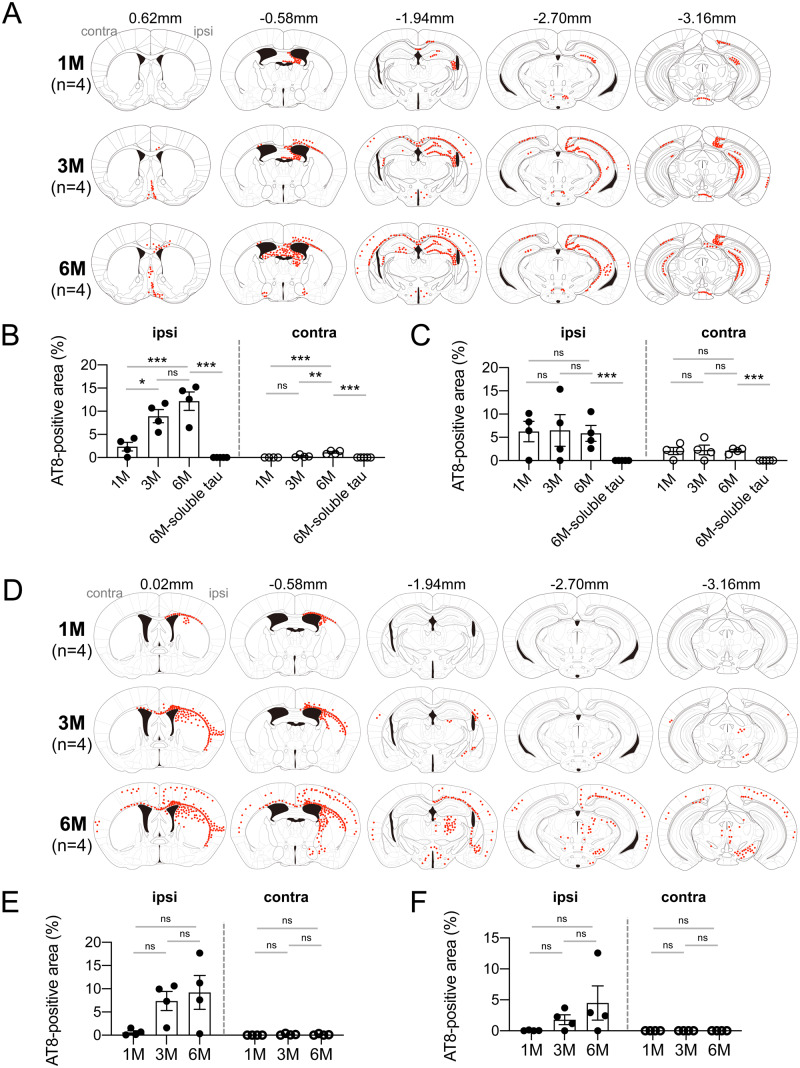
**Distribution of tau pathology in the mice injected with DS-induced murine tau filaments.** (**A**) Distribution of AT8-positive staining (in red) 1, 3 and 6 months (M) after unilateral injection into the hippocampus. (**B**) Quantification of AT8-positive area in the hippocampus after injection into the hippocampus. (**C**) Quantification of AT8-positive area in the mammillary nucleus after injection into the hippocampus. (**D**) Distribution of AT8-positive staining 1, 3 and 6 months after unilateral injection into the striatum. (**E**) Quantification of AT8-positive area in the corpus callosum and striatum after injection into the striatum. (**F**) Quantification of AT8-positive area in the substantia nigra after injection into the striatum. Mean and SEM are shown in the graph. Statistical analysis was performed using one-way ANOVA with Tukey’s *post hoc* test (*P < 0.05; **P < 0.01; ***P < 0.001).

Distribution of AT8-positive staining in the brains of wild-type mice at 1, 3 and 6 months after injection of DS-induced murine tau assemblies into the hippocampus and the striatum is summarized in Fig. 6. The pattern of distribution was dependent on the injection sites; injection into hippocampus-induced pathology mainly in the hippocampus, fimbria-fornix, septal nucleus, mammillary nucleus and entorhinal cortex (Fig. 6A). Quantitative analyses showed that AT8-positive area increased in a time-dependent manner in both the injected and contralateral hemispheres of hippocampus (Fig. 6B), while in the mammillary nucleus, AT8-positive staining was already observed after 1 month in both hemispheres and it was unchanged over time (Fig. 6C).

Injection into the striatum caused tau spreading in the substantia nigra, amygdala and some cortical areas (Fig. 6D). AT8-positive area appears to increase over time both in the striatum and corpus callosum (Fig. 6E), and the substantia nigra (Fig. 6F) in the injected hemisphere; however, no significant differences were observed (*P* > 0.05).

Furthermore, injection of DS-induced murine and human tau seeds into the hippocampus showed the similar patterns of distribution (Fig. 6A and Supplementary Fig. 5A). In the mice injected with human tau seeds, AT8-positive pathology showed a tendency to increase over time in the ipsilateral hippocampus (Supplementary Fig. 5B) and in the both sides of mammillary nucleus (Supplementary Fig. 5C). Comparison of AT8-positive area between the mice injected with mouse tau seeds and those injected with human tau seeds is shown in Supplementary Table 2. In the ipsilateral hippocampus, the mice injected with mouse tau seeds had significantly more AT8-positive staining at 1 and 3 months after injection (*P* = 0.0453 and *P* = 0.0442, respectively), while at 6 months, there was no significant difference between them. In the contralateral hippocampus, no significant difference was observed. In the mammillary nucleus, at 1 month after injection, the mice injected with murine tau seeds had significantly more AT8-positive pathology in both hemispheres (*P* = 0.0298 for the ipsilateral hemisphere and *P* = 0.0362 for the contralateral side), however, from 3 months onward, there was no difference in AT8-positive tau pathology (*P* > 0.05).

### DS-induced seeds cause formation of sarkosyl-insoluble tau after injection in the brain

**Figure 7:**
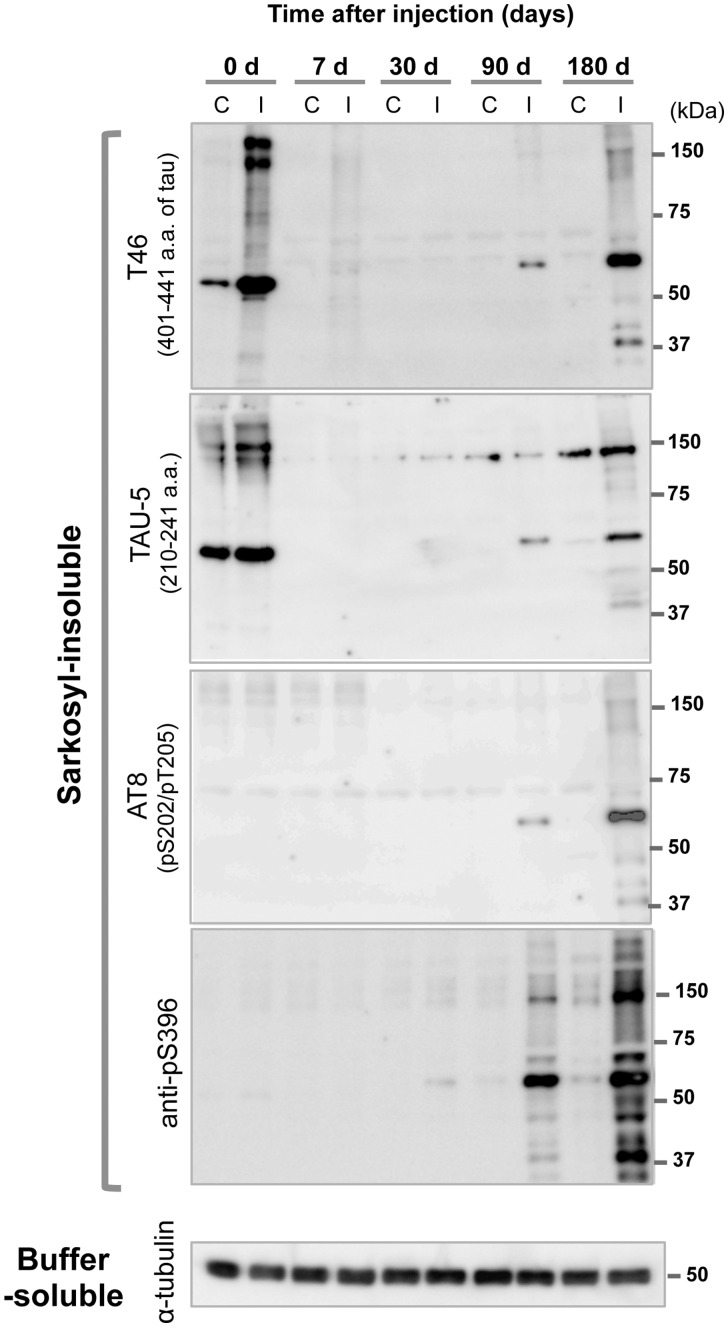
**Biochemical analysis of the brains in wild-type mice after unilateral injection of DS-induced murine tau filaments into the hippocampus.** Sarkosyl-insoluble fractions were prepared 0, 7, 30, 90 and 180 days after injection and analysed by western blotting with anti-tau antibodies T46, TAU-5, AT8 and anti-pS396. Western blots of the sarkosyl-soluble fraction with anti-α-tubulin as the loading control. Full-length blot is shown in Supplementary Fig. 7. I = ipsilateral (injected side); C = contralateral.

The effects of injection of recombinant murine tau assembled into filaments using DS into brains of wild-type mice were also analysed biochemically. Brains were collected at 0, 7, 30, 90 and 180 days after injection. At Day 0, sarkosyl-insoluble tau was detected with T46 and TAU-5 antibodies on the ipsilateral side, while small amount of that was detected on the contralateral side (Fig. 7). The major tau band had an apparent molecular mass of 55 kDa. In view of the latter, the absence of reactivity with phosphorylation-dependent anti-tau antibodies (AT8 and anti-pS396), it appears likely that this staining was of the injected assemblies. The disappearance of labelling by Day 7 indicates that the injected assemblies were degraded in a week, as observed in mice injected with synthetic tau seeds (Peeraer *et al.*, 2015) and α-synuclein fibrils (Masuda-Suzukake *et al.*, 2013). At 90 days, a sarkosyl-insoluble band was detected by T46 and TAU-5 on the ipsilateral side, which increased in intensity by Day 180 (Fig. 7). This material was also positive for AT8 and anti-pS396 at 90 days, with a stronger signal at 180 days. Staining with anti-pS396 was already seen at 30 days on the ipsilateral side. Compared with the sarkosyl-insoluble tau bands at Day 0, the mobility of bands present from Day 30 onwards was reduced; they ran at ∼64 kDa, suggesting that they were hyperphosphorylated.

## Discussion

We show here that intracerebral injection of DS-induced tau assemblies seeded aggregation and allowed propagation of inclusions in wild-type mice. This is similar to what was described for human brain extracts in wild-type mice (Lasagna-Reeves *et al.*, 2012; Audouard *et al.*, 2016; Guo *et al.*, 2016; Narasimhan *et al.*, 2017; Ferrer *et al.*, 2019*a*, *b*; Vergara *et al.*, 2019). However, the usage of human brain extracts might have some limitations; there is a variation of pathological tau among cases in terms of the quality (i.e. post-translational modifications) and the quantity (amount of insoluble tau), human samples include other insoluble components such as amyloid β that might work as a promoting factor for tau accumulation (Gotz *et al.*, 2001; Vasconcelos *et al.*, 2016), and regulation of tissue availability. Previous reports have also shown that intracerebral injection of synthetic tau filaments prepared from recombinant mutant tau (P301L or P301S) with a Myc tag comprised of the four microtubule-binding repeats of tau (K18), or full-length protein, induced spreading of tau pathology in transgenic mice overexpressing mutant human tau (P301L or P301S) (Iba *et al.*, 2013; Peeraer *et al.*, 2015; Stancu *et al.*, 2015, 2019), but not in non-transgenic mice (Peeraer *et al.*, 2015). Accumulation of endogenous tau has been reported in tau transgenic mice after injection of other amyloidogenic proteins: insoluble amyloid β or alpha-synuclein (Gotz *et al.*, 2001; Guo *et al.*, 2013; Castillo-Carranza *et al.*, 2018), suggesting that cross-seeding of tau occur *in vivo*, however, it remains unclear whether it takes place in non-transgenic mice.

In this study, *in vitro* analyses revealed that DS-induced tau filaments were shorter than those induced by HP (Fig. 1A and Supplementary Fig. 2A), in confirmation of the previous report (Hasegawa *et al.*, 1997), and gave higher levels of thioflavin fluorescence (Fig. 1B and Supplementary Fig. 2B). Since fragmentation of HP-induced tau filaments by sonication did not change thioflavin fluorescence (Supplementary Fig. 1), factors other than the difference of length between HP- and DS-induced tau filaments may affect the level of fluorescence. The latter may indicate qualitatively different thioflavin binding sites as the result of structural differences between tau filaments formed by HP and the more sulphated DS, as has been reported for α-synuclein filaments (Sidhu *et al.*, 2018). This is also supported by the findings that murine tau in the presence of HP exhibited greater sarkosyl-insolubility than that in the presence of DS (Fig. 1C), and human tau in the presence of HP formed more insoluble aggregates at 168 h (Supplementary Fig. 2C). A decrease in sarkosyl-insoluble tau in the presence of DS at 168 h (Fig. 1C and Supplementary Fig. 2C) suggests that DS-induced tau filaments are more vulnerable to shaking. Preformed filaments by DS showed lower stability with GuHCl treatment (Fig. 1E and Supplementary Fig. 2E), indicating that HP and DS each induce assemblies with distinct structural features. Taken together, it appears likely that DS-induced filaments are more fragile than those induced by HP.

All mice injected with DS-induced murine tau filaments had AT8-positive inclusions at the injection sites from 1 month onward and other phosphorylation-dependent anti-tau antibodies also stained the inclusions (Fig. 3A), which were thioflavin S- and Gallyas-positive (Figs 3B and C and 5C), indicative of cross-β conformation typical of amyloids. Injection of DS-induced human tau filaments induced accumulation and propagation of endogenous mouse tau (Fig. 4C and Supplementary Fig. 3). Induction of tau pathology by human tau seeds was slightly slower than that by mouse tau seeds at the early time points, however, at 6 months after injection, there was no significant difference in AT8-positive area between them (Supplementary Table 2), indicating that human tau seeds cross the species barrier. The absence of a significant species barrier is consistent with previous studies demonstrating seeding by insoluble tau from human tauopathy brains in wild-type mice (Clavaguera *et al.*, 2013; Audouard *et al.*, 2016; Guo *et al.*, 2016; Narasimhan *et al.*, 2017; Ferrer *et al.*, 2019*a*, *b*; Vergara *et al.*, 2019). The amino acid sequences of the structural cores of 4R tau human filaments induced by HP (Zhang *et al.*, 2019) or isolated from the brains of corticobasal degeneration patients are identical between mouse and human (Zhang *et al.*, 2020). Moreover, the sequence of the trypsin-resistant filament core is 99.4% homologous (Taniguchi-Watanabe *et al.*, 2016). However, the structures of seeded 4R tau filaments from mouse brain remain to be determined.

**Figure 8:**
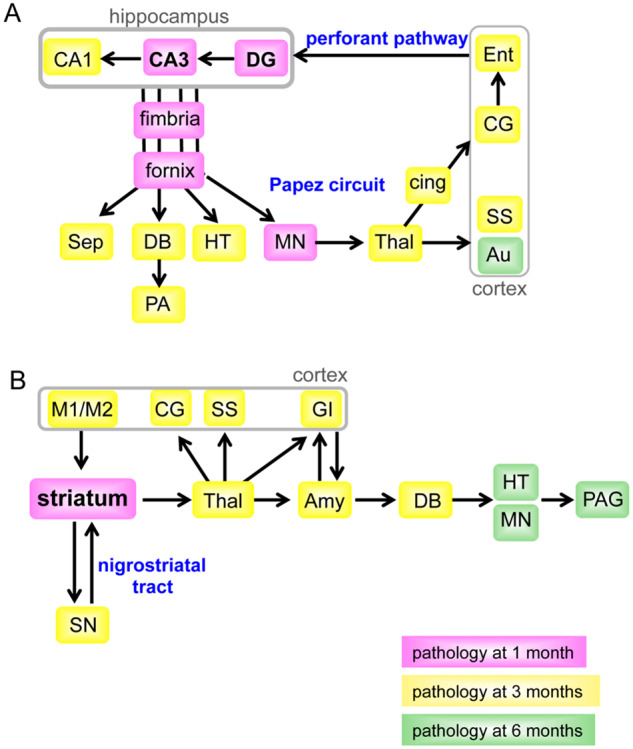
**The regions connected to the hippocampus (**A**) and striatum (**B**) with AT8-positive staining after injection of DS-induced murine tau filaments.** Pink, yellow and green boxes indicate the regions with AT8-positive staining 1, 3 and 6 months after injection. Arrows indicate the direction of projections. Amy = amygdala; Au = auditory cortex; cing = cingulum; CG = cingulate gyrus; DB = nucleus of the diagonal band; DG = dentate gyrus; EC = entorhinal cortex; GI = granular insular cortex; HT = hypothalamus; M1/M2 = primary and secondary motor cortices; MN = mammillary nucleus; PA = pre-optic area; PAG = periaqueductal grey; Sep = septal nucleus; SN = substantia nigra; SS = somatosensory cortex; Thal = thalamus.

To understand better the spread of tau assemblies, we summarize the distribution of tau pathology among the brain regions in Fig. 8A following injection into the hippocampus and in Fig. 8B following injection into the striatum.

One month after injection into the hippocampus, AT8 staining was present in the dentate gyrus, CA3 layer, fimbria/fornix and both hemispheres of mammillary nucleus. Although the mammillary nucleus is located at a distance from the injection site, it receives bilaterally inputs from the hippocampus through the fimbria/fornix (Mathiasen *et al.*, 2019). In addition, 3 months after injection, tau assemblies were present in the CA1 layer, other regions with input from the hippocampus through the fimbria/fornix, including septal nucleus, nucleus of the diagonal band, pre-optic area and hypothalamus, and in some brain regions with connections from the mammillary nucleus, including the thalamus, cingulum, cingulate gyrus, somatosensory cortex and entorhinal cortex. At 6 months after injection, tau pathology was spread to the other cortical areas, such as the auditory cortex.

One month after injection into the striatum (Fig. 8B), AT8 staining was present in striatum and corpus callosum. At 3 months after injection, it was present in the regions projecting from the striatum, including the substantia nigra, thalamus, amygdala and diagonal band nucleus and spread to the motor, somatosensory, cingulate and insular cortices. The motor cortex projects to the striatum, the somatosensory and cingulate cortices receive input from the thalamus, whereas the insular cortex is connected with both the thalamus and amygdala. At 6 months after injection, AT8 staining was spread further to the hypothalamus, mammillary nucleus and periaqueductal grey.

Further studies to evaluate neurodegeneration and behavioural changes in the mice injected with DS-induced tau seeds are in preparation.

Collectively, our findings demonstrate that intracerebral injection of DS-induced murine and human tau seeds in wild-type mice caused pathology at the injection sites that spread in a time-dependent manner both anterogradely and retrogradely to distant areas through the neuronal projections.

## Supplementary material

Supplementary material is available at *Brain Communications* online.

## Supplementary Material

fcaa091_Supplementary_Data

## Abbreviations

Amy =: amygdala
Au =: auditory cortex
cc =: corpus callosum
CG =: cingulate gyrus
Cing =: cingulum
contra =: contralateral hemisphere
DB =: nucleus of the diagonal band
DG =: dentate gyrus
dhc =: dorsal hippocampal commissure
DS =: dextran sulphate
DTT =: dithiothreitol
Ent =: entorhinal cortex
f =: fornix
fi =: fimbria
GI =: granular insular cortex
GuHCl =: guanidine hydrochloride
Hippo =: hippocampus
HP =: heparin
HT =: hypothalamus
ipsi =: ipsilateral hemisphere
M1/M2 =: primary and secondary motor cortices
MN =: mammillary nucleus
Mr =: molecular weight marker
PA =: pre-optic area
PAG =: periaqueductal grey
SEM =: standard error of the mean
Sep =: septal nucleus
SN =: substantia nigra
SS =: somatosensory cortex
Str =: striatum
Thal =: thalamus
