# Supplementary material for: Dextran sulphate-induced tau assemblies cause endogenous tau aggregation and propagation in wild-type mice
Source: Brain Commun. 2020 Jul 8;2(2):fcaa091. doi: 10.1093/braincomms/fcaa091 (PMC7519727; doi:10.1093/braincomms/fcaa091)
Supplement: fcaa091_Supplementary_Data [file fcaa091_supplementary_data.pdf]

Supplementary Table 1

Primary antibodies used in this study.

	epitope	*host**	*supplier*	*cat#*	*dilution***
AT8	pS202/pT205	Ms	ThermoFisher	MN1020	IHC: 1/1000<br>IF: 1/200<br>WB: 1/1000
AT100	pT212/pS214/pT217	Ms	ThermoFisher	MN1060	IHC: 1/100
AT180	pT231	Ms	ThermoFisher	MN1040	IHC: 1/500
12E8	pS262 and/or pS356	Ms	Elan Pharmaceuticals	-	IHC: 1/100
PHF-1	pS396/pS404	Ms	gift from Dr. Peter Davis	-	IHC: 1/500
anti-pS396	pS396	Rb	ThermoFisher	44-752G	IHC: 1/500<br>IF: 1/500<br>WB: 1/2000
anti-pS422	pS422	Ms	Wako	016-27681	IHC: 1/500
anti-mouse tau	114-127 a.a. of mouse tau	Rb	In-house	-	IF: 1/100
T46	401-441a.a. of tau	Ms	ThermoFisher	13-6400	WB: 1/3000
Tau5	210-241a.a. of tau	Ms	Calbiochem	577801	WB: 1/2000
alpha-tubulin	alpha-tubulin	Ms	SIGMA	T6199	WB: 1/5000
NeuN	NeuN	Rb	Abcam	ab177487	IF: 1/200
p62	p62/sequestosome 1	Guinea pig	Progen	GP62-C	IF: 1/500
Tuj1	neuron specific class III beta-tubulin	Rb	Abcam	ab18207	IF: 1/500
Ub	ubiquitin	Ms	MBL	MK-11-3	IF: 1/500

* Ms: mouse, Rb: rabbit

** IHC: immunohistochemistry, IF: immunofluorescence, WB: western blotting

Supplementary Table 2

Comparison of AT8-positive area at 1, 3, 6 months after injection between the mice injected with mouse tau seeds and those injected with human tau seeds.
Mean values of AT8-positive area (%) in the hippocampus (injection site) and the mammillary nucleus from 4 mice/group are shown. Statistical analysis was performed with unpaired t-test.
ipsi=ipsilateral (injected hemisphere); contra=contralateral hemisphere.

Brain region analysed		Time after injection	Injection materials		P values
			**Mouse** tau seeds (AT8-positive area, %)	**Human** tau seeds (AT8-positive area, %)	
Hippocampus	ipsi	1M	2.35	0.03	* p=0.0453
		3M	8.93	3.92	* p=0.0442
		6M	12.19	7.52	ns
	contra	1M	0	0	ns
		3M	0.28	0.18	ns
		6M	1.12	0.61	ns
Mammillary nucleus	ipsi	1M	6.24	0	* p=0.0298
		3M	6.49	1.27	ns
		6M	5.86	3.78	ns
	contra	1M	2.06	0	* p=0.0362
		3M	2.28	0.88	ns
		6M	2.11	1.37	ns

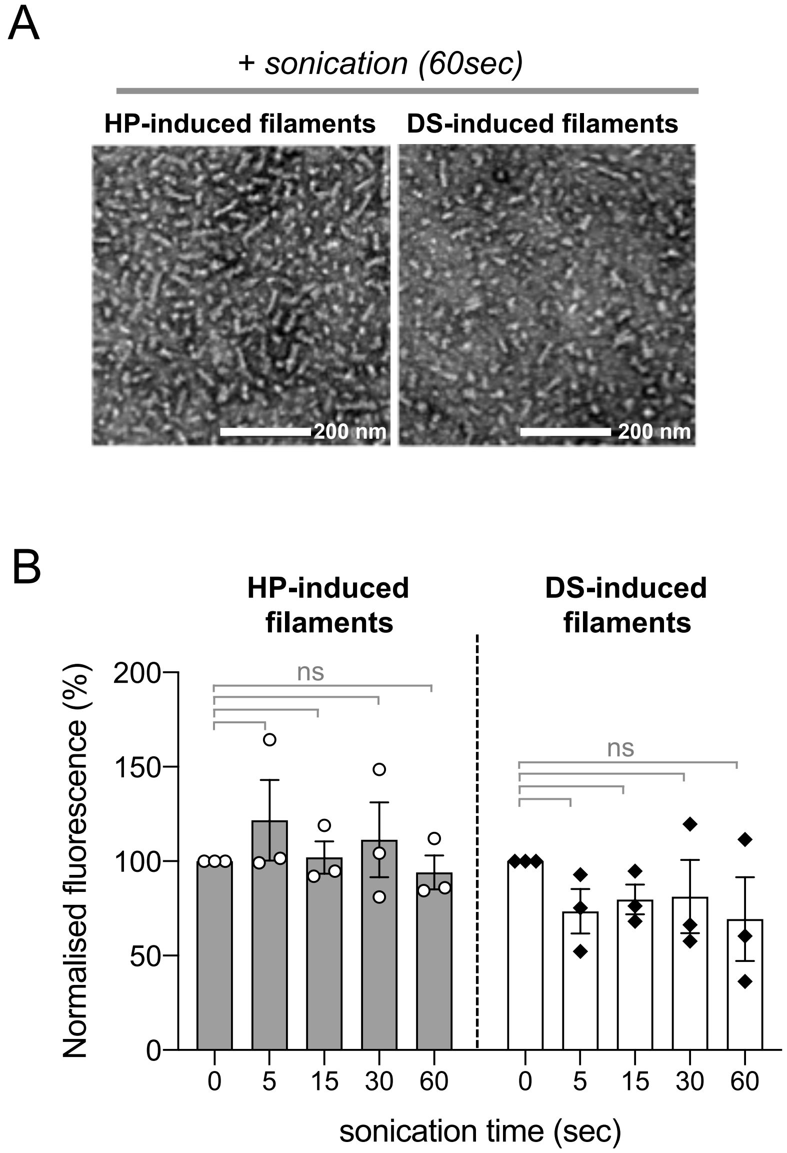

Supplementary Figure 1

Fragmentation of HP- and DS-induced murine tau filaments has no effect on the level of thioflavin fluorescence.

Preformed tau filaments were sonicated using a Sonifier SFX250 cup horn sonicator (BRANSON). (A) Negative stain electron micrographs of HP- and DS-induced tau filaments after 60 sec sonication. Scale bar, 200 nm. (B) No significant change of thioflavin fluorescence was observed in both HP- and DS-induced filaments after fragmentation by sonication. Data are shown as percentage relative to the fluorescence values measured before sonication. Mean ± S.E.M are shown in the graph (n=3). Statistical analysis was performed with one-way ANOVA with Tukey’s post hoc test.

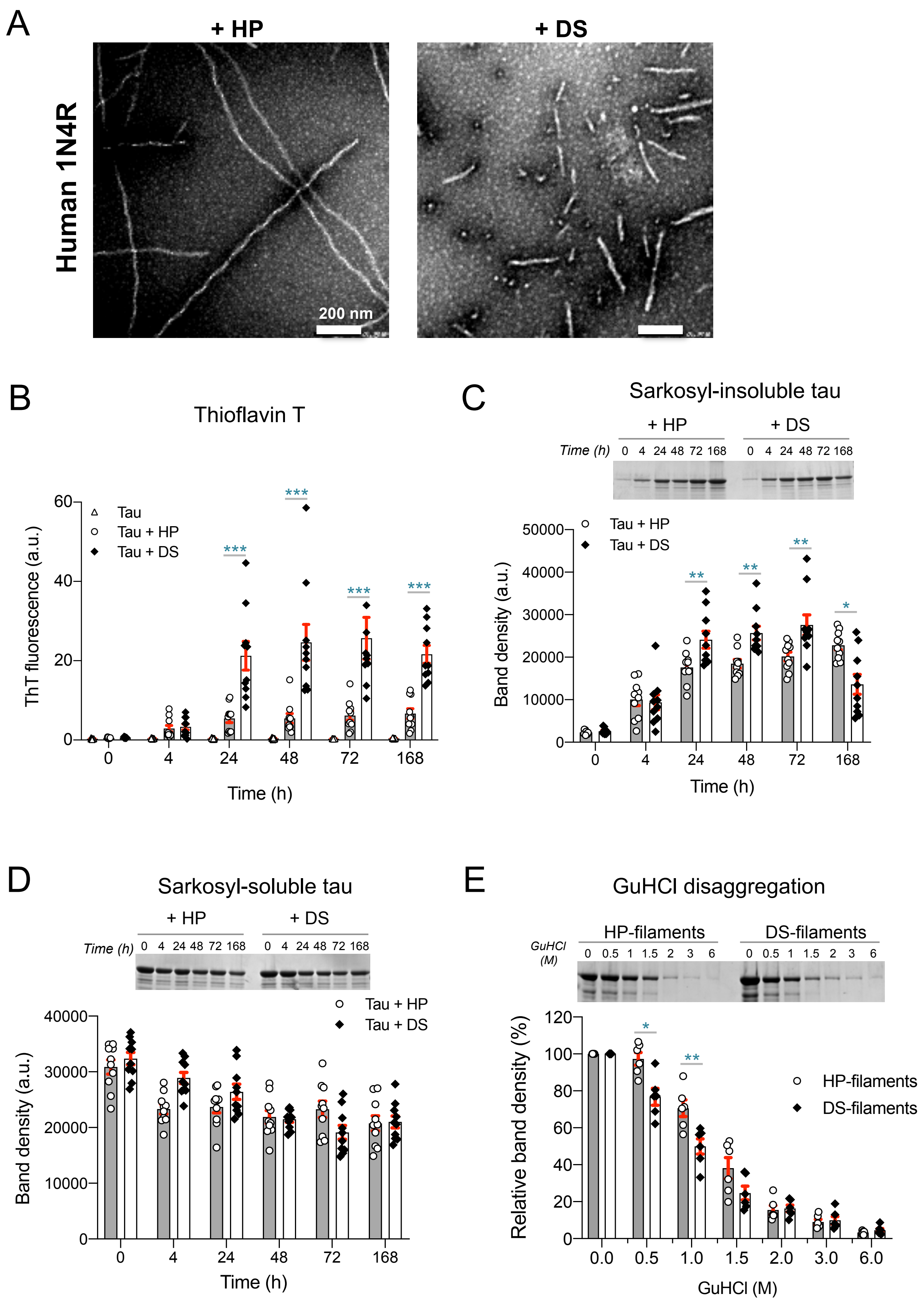

Supplementary Figure 2

Supplementary Figure 2

Different properties of HP- and DS-induced human tau filaments.
(A) Negative stain electron micrographs of recombinant human 1N4R tau following incubation with heparin (HP) or dextran sulphate (DS). (B) Kinetics of tau assembly in the presence of HP or DS based on thioflavin T fluorescence (n=10). (C,D) Formation of sarkosyl-insoluble and -soluble tau in the presence of HP or DS. The proteins were separated on SDS-PAGE, stained with CBB and quantified by ImageJ software (n=10). Full-length gel images are shown in Supplementary Fig. 6D-E. (E) Guanidine hydrochloride disaggregation of preformed human tau filaments. HP-induced tau filaments are more resistant to low concentration (0.5-1 M) of guanidine hydrochloride (GuHCl) than DS-induced tau filaments (n=6). Full-length gel image is shown in Supplementary Fig. 6F. Mean and S.E.M are shown in the graph. Statistical analysis was performed with unpaired t-test (*$p<0.05$; **$p<0.01$; ***$p<0.001$).

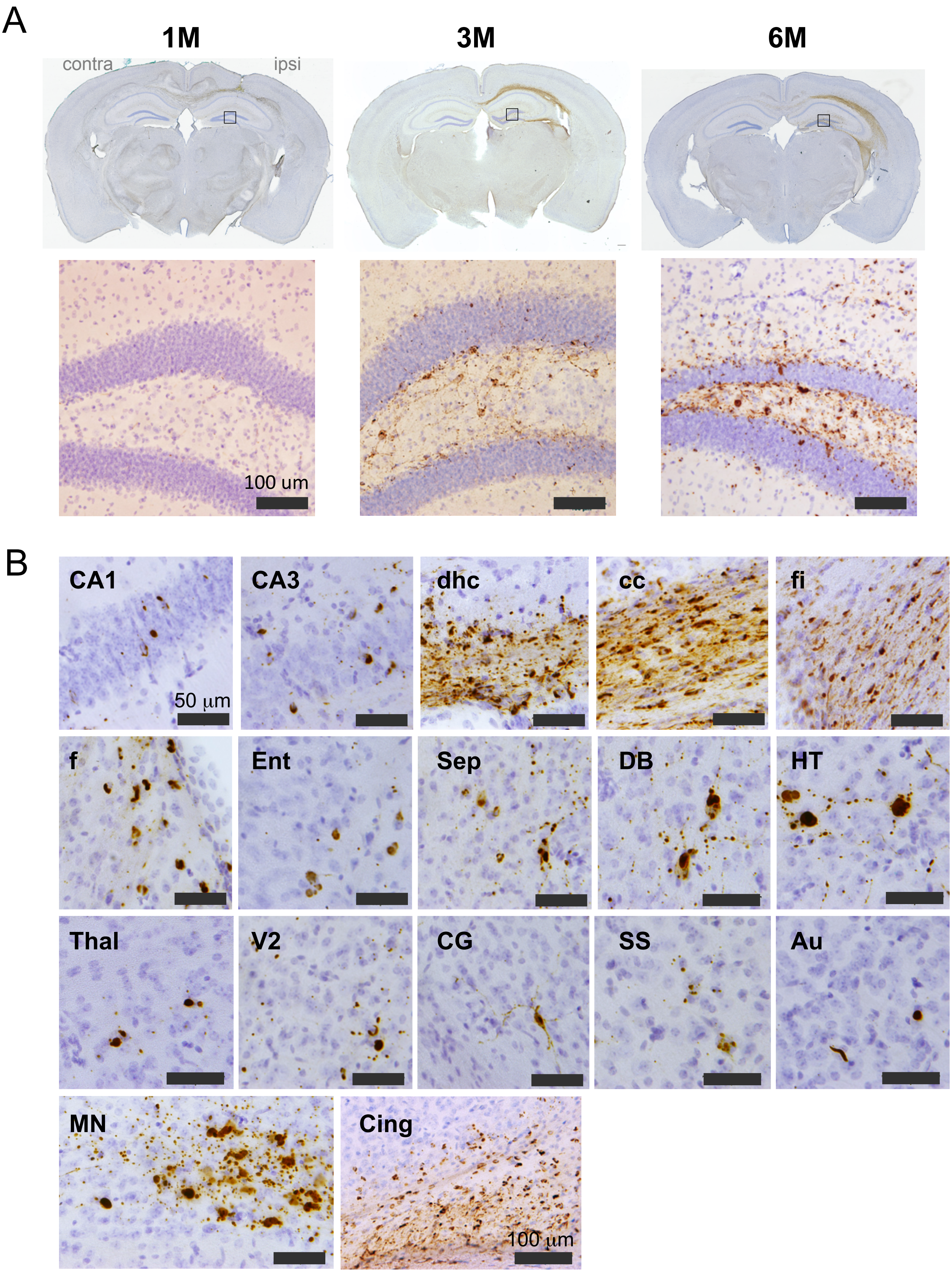

Supplementary Figure 3

Supplementary Figure 3

AT8-positive staining after injection of DS-induced human tau filaments into the hippocampus of wild-type mice.

(A) AT8 staining after 1, 3 and 6 months (M) of injection. Higher magnification of the boxed areas is shown. (B) AT8 staining at 6 M after injection.

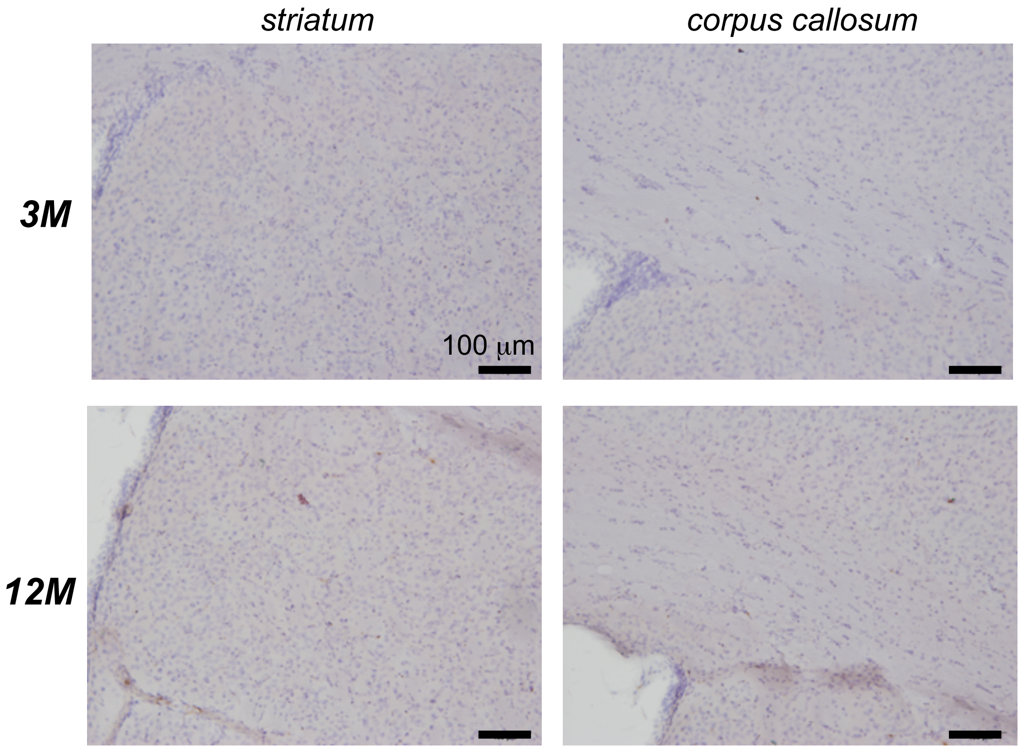

Supplementary Figure 4

AT8-positive staining is not observed after injection of DS-induced murine tau filaments into the striatum of tau knockout mice.
Brain sections at 3 and 12 months (M) after injection were stained with AT8.

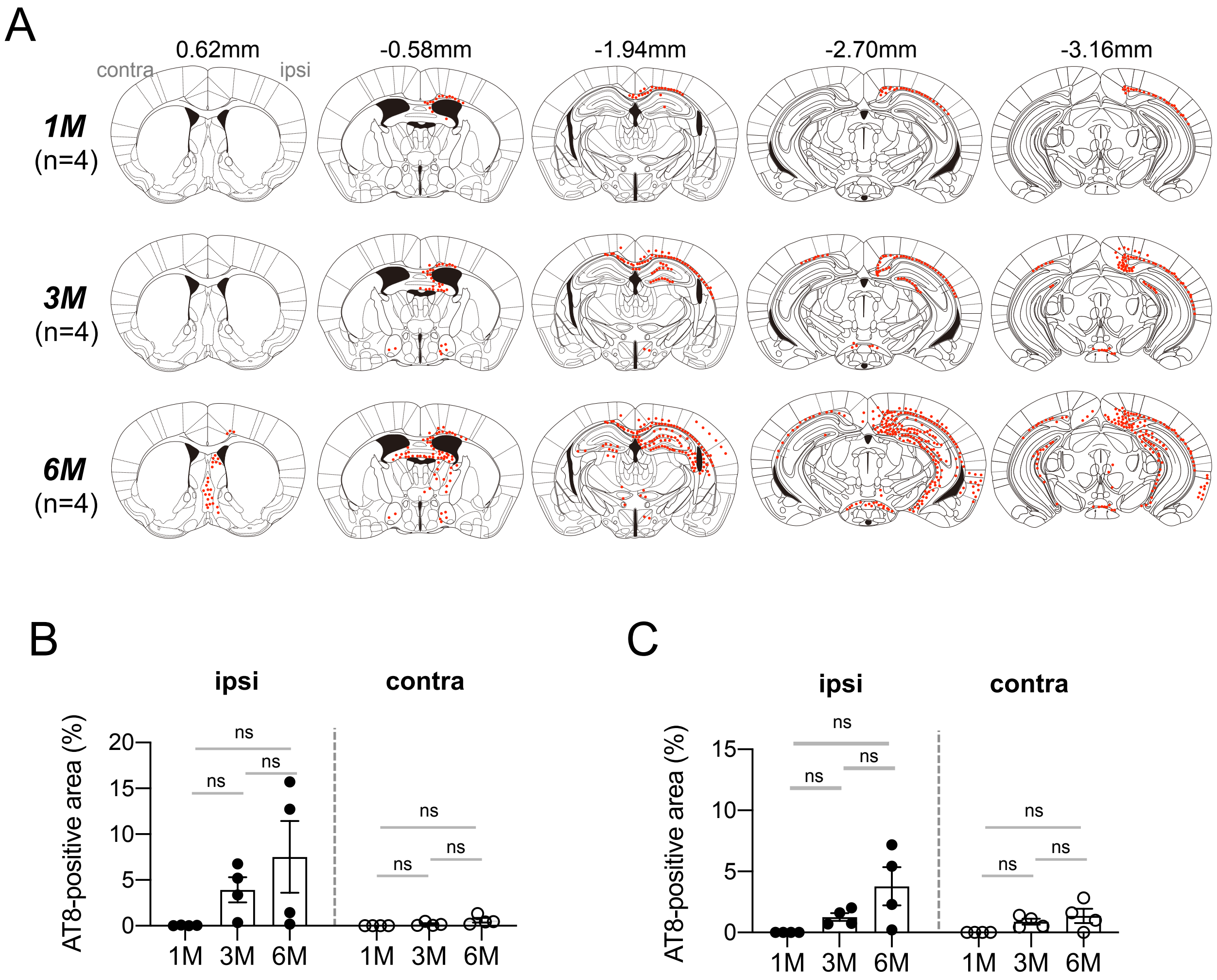

Supplementary Figure 5

Distribution of tau pathology in the mice injected with DS-induced human tau filaments into the hippocampus of wild-type mice.

(A) Distribution of AT8-positive staining (in red) 1, 3, and 6 months (M) after unilateral injection into the hippocampus. (B) Quantification of AT8-positive area in the hippocampus. (C) Quantification of AT8-positive area in the mammillary nucleus. Mean and S.E.M. are shown in the graph. Statistical analysis was performed using one-way ANOVA with Tukey's post hoc test.

ipsi=ipsilateral (injected) hemisphere; contra=contralateral hemisphere.

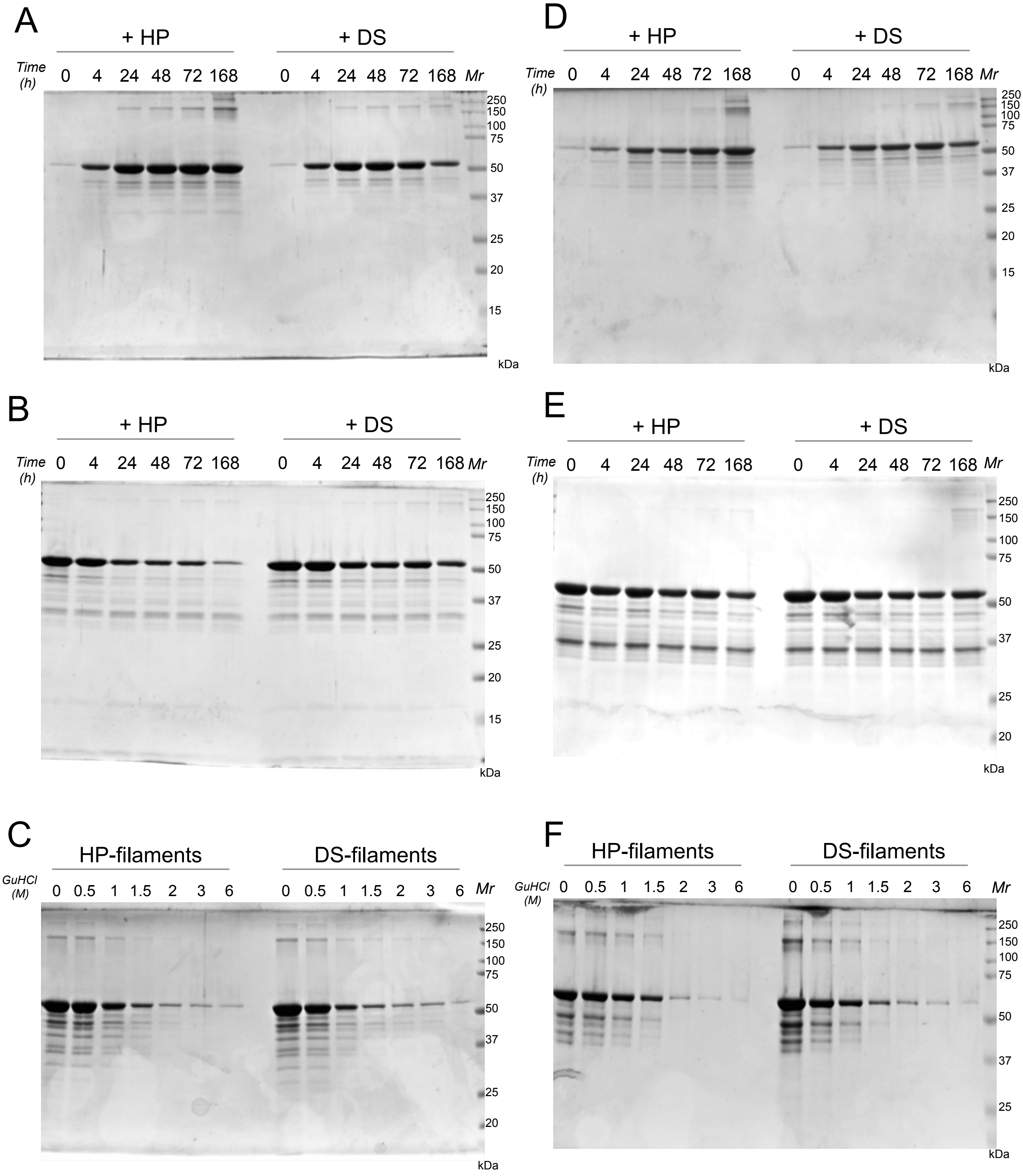

Supplementary Figure 6

Full-length gel images.

(A) Full-length gel for Fig. 1C. (B) Full-length gel for Fig. 1D. (C) Full-length gel for Fig. 1E. (D) Full-length gel for Supplementary Fig. 2C. (E) Full-length gel for Supplementary Fig. 2D. (F) Full-length gel for Supplementary Fig. 2E.

Mr=Molecular weight marker.

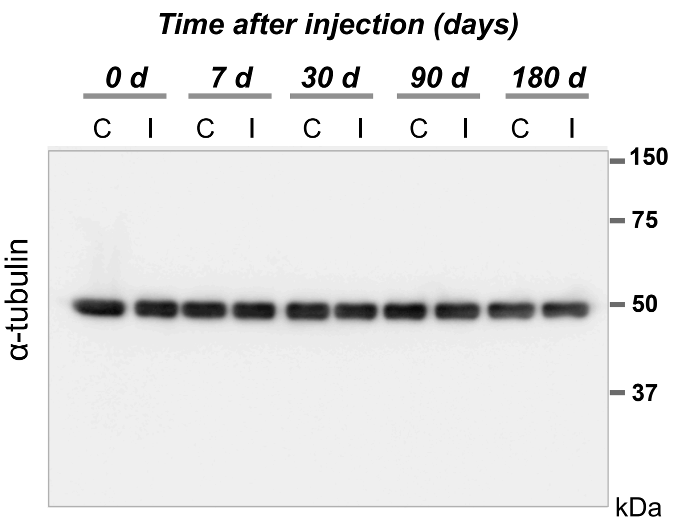

Supplementary Figure 7

Full-length blot image stained with α-tubulin antibody for Figure 7.
